# Evaluating the effects of air cushions on body pressure distribution and thermal insulation in evacuation shelters: A randomized controlled crossover study

**DOI:** 10.1371/journal.pgph.0005259

**Published:** 2025-10-06

**Authors:** Seiji Hamanishi, Shinsuke Sasaki

**Affiliations:** 1 Graduate School of Nursing/Nursing Faculty, Kansai University of Social Welfare, Ako, Hyogo, Japan; 2 Faculty of Health and Welfare Science, Okayama Prefectural University, Soja, Okayama, Japan; PLOS: Public Library of Science, UNITED STATES OF AMERICA

## Abstract

In large-scale disasters such as the anticipated Nankai Trough Earthquake, millions of evacuees are expected to remain in shelters, often sleeping on cold, hard floors in classrooms and gymnasiums, contributing to musculoskeletal pain and sleep disruption. While cardboard beds have been employed since the Great East Japan Earthquake due to their rapid mass-production capability, municipalities are not required to stockpile them, and many were unable to provide sufficient quantities during the Kumamoto Earthquake. Furthermore, the large size and weight of these beds complicate timely delivery, and their use alone provides only limited body pressure distribution. Consequently, there is concern that large-scale disasters will result in a severe shortage of appropriate bedding. This study aimed to determine whether air cushions can provide sufficient body pressure distribution and thermal insulation to serve as practical floor mats in evacuation shelters. Twenty healthy adults were enrolled in this randomized controlled crossover trial. Participants were instructed to lie on an air cushion, a urethane pad, and a plastic sheet, during which body pressure distribution was evaluated. Subjective perceptions of firmness and comfort were assessed using a numeric rating scale, and thermal insulation properties were evaluated by measuring surface temperature changes when each material was placed over a cooling gel pack. Compared with plastic sheets, the air cushion reduced mean body pressure by over 20% and increased contact area by more than 30%, with improvements exceeding those observed for urethane pads. Participants rated air cushions as significantly less firm and more comfortable than urethane pads. The air cushion also demonstrated thermal insulation comparable to urethane pads. These findings suggest that air cushions could serve as practical emergency floor mats in evacuation shelters, complementing cardboard beds and potentially reducing musculoskeletal strain and sleep disturbances in disaster settings.

## Introduction

Poor conditions in evacuation shelters have been considered a significant contributor to health problems and disaster-related deaths among evacuees, with concerns intensifying in large-scale disasters such as the predicted Nankai Trough Earthquake [1,2]. According to the Japanese government, approximately 8.8 million people are expected to evacuate in the event of a Nankai Trough Earthquake—about 19 times the number of evacuees during the Great East Japan Earthquake [3]. One of the major challenges during such large-scale disasters is providing sufficient and appropriate bedding for numerous evacuees.

Since cardboard beds can be mass-produced quickly, they have been used as emergency beds since the Great East Japan Earthquake [4]. However, according to the evacuation shelter guidelines, Japanese municipalities are not required to stockpile emergency bedding other than blankets, so cardboard beds are rarely stockpiled by municipalities [5].

In addition, these beds are large and heavy, so it is difficult to deliver them to affected areas promptly. As a result, evacuees are often forced to sleep directly on the floors of classrooms and gymnasiums for weeks or even months following a disaster [6]. It has been suggested that the stress induced by firm and cold floor surfaces in evacuation shelters could disrupt evacuees’ sleep [7–10]. However, during the Kumamoto Earthquake, approximately 60% of municipalities could not provide cardboard beds to those in need [5]. When a large-scale disaster such as the Nankai Trough Earthquake occurs, it is anticipated that many municipalities will face similar difficulties, resulting in an even more severe shortage of bedding [11].

In Japan, school classrooms and gymnasiums commonly serve as evacuation facilities, so the floor mats on which evacuees lie must fulfill two essential functions: blocking cold from the floor and distributing body pressure. Floor mats for shelters also need to be low in cost and compact for storage. Since bubble wrap used for packaging is lightweight, water-resistant, and widely available, we examined its potential as emergency floor mats in our previous study. However, our results indicated that bubble wrap was unable to distribute body pressure adequately because air could not move within its small sealed pockets [8]. In contrast, air cushions with larger cells may allow the interior air to move in response to pressure, potentially improving body pressure distribution [8,12]. Since cardboard beds alone provide limited body pressure dispersion, using an appropriate mattress is recommended to prevent back pain and sleep disorders. After cardboard beds are supplied, these air cushions could then serve as mattresses.

Given these points, this study aimed to determine whether air cushions can provide sufficient body pressure dispersion for use as floor mats in evacuation shelters.

## Materials and methods

### Study subjects

In this study, the required sample size was determined using G*Power (Heinrich-Heine-Universität Düsseldorf, Düsseldorf, Germany). Based on preliminary study data, an effect size of 0.7, an α level of 0.05, and a power (1-β) of 0.80 were assumed, which yielded a calculated sample size of 16. To account for potential dropouts during the study, a total of 20 participants were recruited between September 1 and November 10, 2023. Healthy volunteers aged between 18 and 59 years were recruited through our website, and a total of 20 participants were enrolled in the study. The inclusion criteria were: (1) age between 18 and 59 years; and (2) ability to provide informed consent. The exclusion criteria were: (1) difficulty in assuming the postures required for the experiment; (2) body mass index (BMI) less than 18 or greater than 30; and (3) individuals experiencing severe body pain.

### Study design and protocol

**Fig 1:**
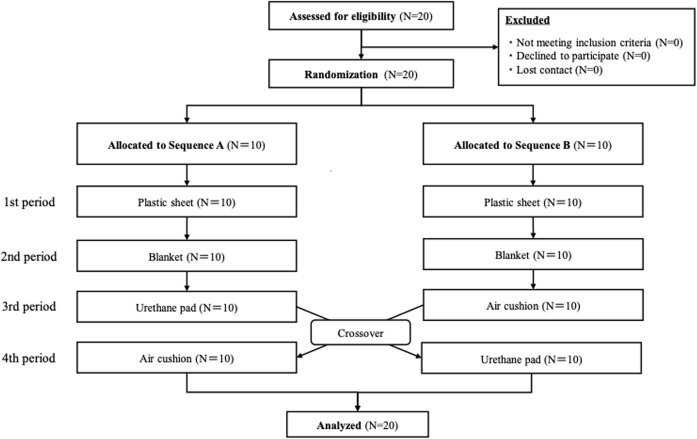
Consort flow diagram.

This randomized controlled crossover trial was conducted at Kansai University of Social Welfare from October through December 2023. Participants were randomly assigned to two sequences (A or B) of 10 individuals each. We generated random numbers using Microsoft Excel (Microsoft, USA). In this study, we selected urethane camping pads and air cushions as candidates for floor mats in evacuation shelters. Subjects were asked to lie on two kinds of floor mats in different orders for each sequence. Additionally, plastic sheets and emergency blankets, which are commonly used in evacuation shelters, were adopted as negative controls. A five-minute washout period was included between each experimental period to minimize carryover effects (Fig 1). We determined the washout period based on the time required for data stabilization as indicated in the guidelines for interface pressure mapping, as well as on the results of our previous research [8,13].

### Floor mats

**Fig 2:**
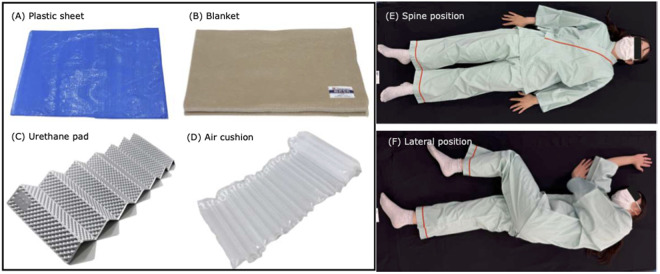
Illustration of the experimental conditions.

We examined whether it would be possible to disperse the body pressure of evacuees by laying potential floor mats on the floors of school classrooms that are commonly used as evacuation shelters. We also compared the body pressure dispersion capacities of two types of mats: an air cushion (CAPZUEE, China) and a urethane pad (Mozambique, Japan). These materials are shown in Fig 2.

### Insulation capability of floor mats

In this study, we evaluated the insulating effects of four types of mats using a thermistor-based thermometer (Model AD-5657-50; A&D Company, Japan). An ice pack was placed in contact with the backside of the mats to which sensors were attached, and temperature changes were recorded over a 10-minute period. To minimize the influence of ambient temperature, the sensors were covered with Styrofoam.

### Body pressure distributions

While participants lay on each mat in the supine and lateral positions, the average body pressure and contact contour area were measured using a body pressure distribution measurement system (BodiTrak Pro 2: Vista Medical, Canada). Body pressure measurements were conducted after participants lay down, allowing sufficient time for the values to stabilize. To ensure data reliability, body pressure was measured twice for each participant in the same position, and the average value was calculated for analysis.

### Subjective evaluation

Subjective comfort and perceived firmness were assessed using a Numerical Rating Scale (NRS). After measuring body pressure distribution, participants rated both their comfort and perceived firmness on a scale from 0 to 10.

### Data analysis

In this study, a linear mixed-effects model was used to compare the body pressure distribution capabilities of various floor mats. Period, sequence, and bedding material were entered into the model as fixed effects, and participants were entered as random effects. Age, sex, and BMI were included as covariates to control for potential confounding factors. A Bonferroni correction was applied for multiple comparisons among mat materials. In our study, the effect size for the linear mixed-effect models were measured using R^2 [14]. Statistical significance was set at a p-value of less than 0.05. Data were analyzed using IBM SPSS Statistics for Windows, version 28.0 (IBM, USA).

### Ethics

This study was conducted in accordance with the Declaration of Helsinki and was approved by the Ethics Review Committee of Kansai University of Social Welfare (Approval No.: 5–0845). Written informed consent was obtained from all participants prior to their inclusion in the study. The study was registered in the University Hospital Medical Information Network Clinical Trials Registry (Registration No. UMIN000051487, https://center6.umin.ac.jp/cgi-open-bin/ctr_e/ctr_view.cgi?recptno=R000058741).

## Results

**Fig 3:**
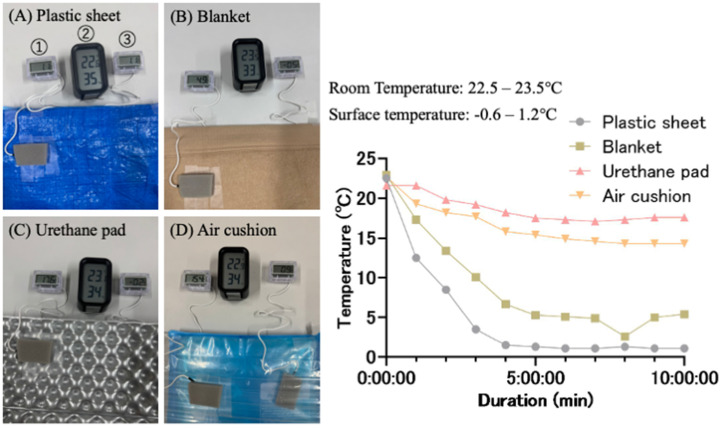
Change in surface temperature. Surface temperature changes of each material in contact with the ice pack were measured using a thermistor-based thermometer. To minimize the influence of ambient temperature, the sensor probe (1) was covered with urethane. The surface temperature of the ice pack (3) and the ambient temperature (2) were also measured simultaneously.

Participant characteristics are presented in Table 1. The participants were predominantly female (95.0%), with a mean age of 23.5 years and a mean BMI of 20.3 kg/m^2^. Fig 3 displays the results of the insulation experiment using four different types of mats: plastic sheets, blankets, urethane pads, and air cushions. Before cooling, the surface temperatures of all mats were nearly identical to the room temperature. After contact with the ice pack, their surface temperatures gradually decreased and stabilized after approximately 10 minutes. The plastic sheet’s surface temperature dropped to nearly the same level as that of the ice pack, whereas the blanket’s temperature remained about 5°C higher. The urethane pad’s surface temperature did not fall below approximately 17°C, and the air cushion’s temperature remained around 15°C. Throughout the experiment, the ice pack’s surface temperature ranged from -0.6°C to 1.2°C.

**Table 1:** Characteristics of Participants.

	Sequence A (N = 10)	Sequence B (N = 10)
Sex (Male / Female)	0 / 10	1 / 9
Age	20.30 ± 1.06	26.70 ± 11.57
BMI	19.92 ± 2.06	20.62 ± 1.00

Age and BMI are presented as Mean ± SD.

**Fig 4:**
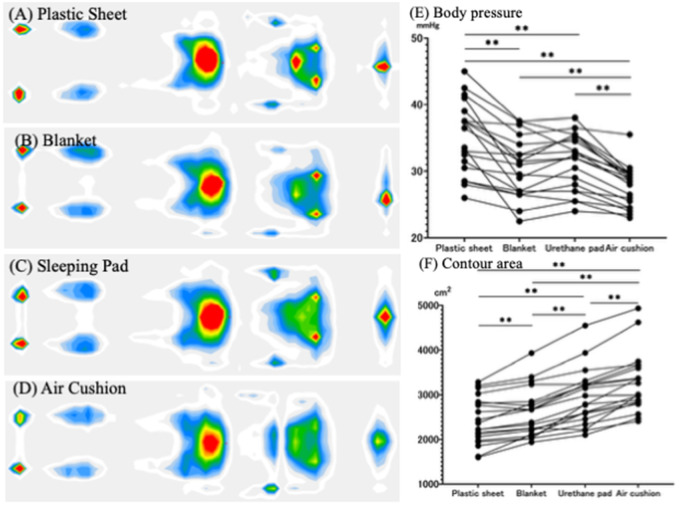
Body Pressure Distribution_(Supine Position). Each dot represents an individual data measurement, and lines connecting these dots indicate repeated measurements from the same participant. All data were compared using a Linear Mixed Effect Model. * P < 0.05, ** P < 0.01.

**Fig 5:**
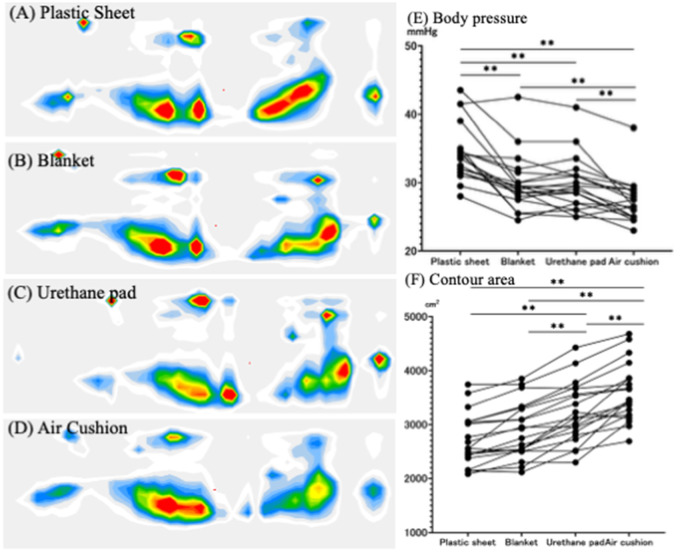
Body Pressure Distribution_(Lateral Position). Each dot represents an individual data measurement, and lines connecting these dots indicate repeated measurements from the same participant. All data were compared using a Linear Mixed Effect Model. * P < 0.05, ** P < 0.01.

**Fig 6:**
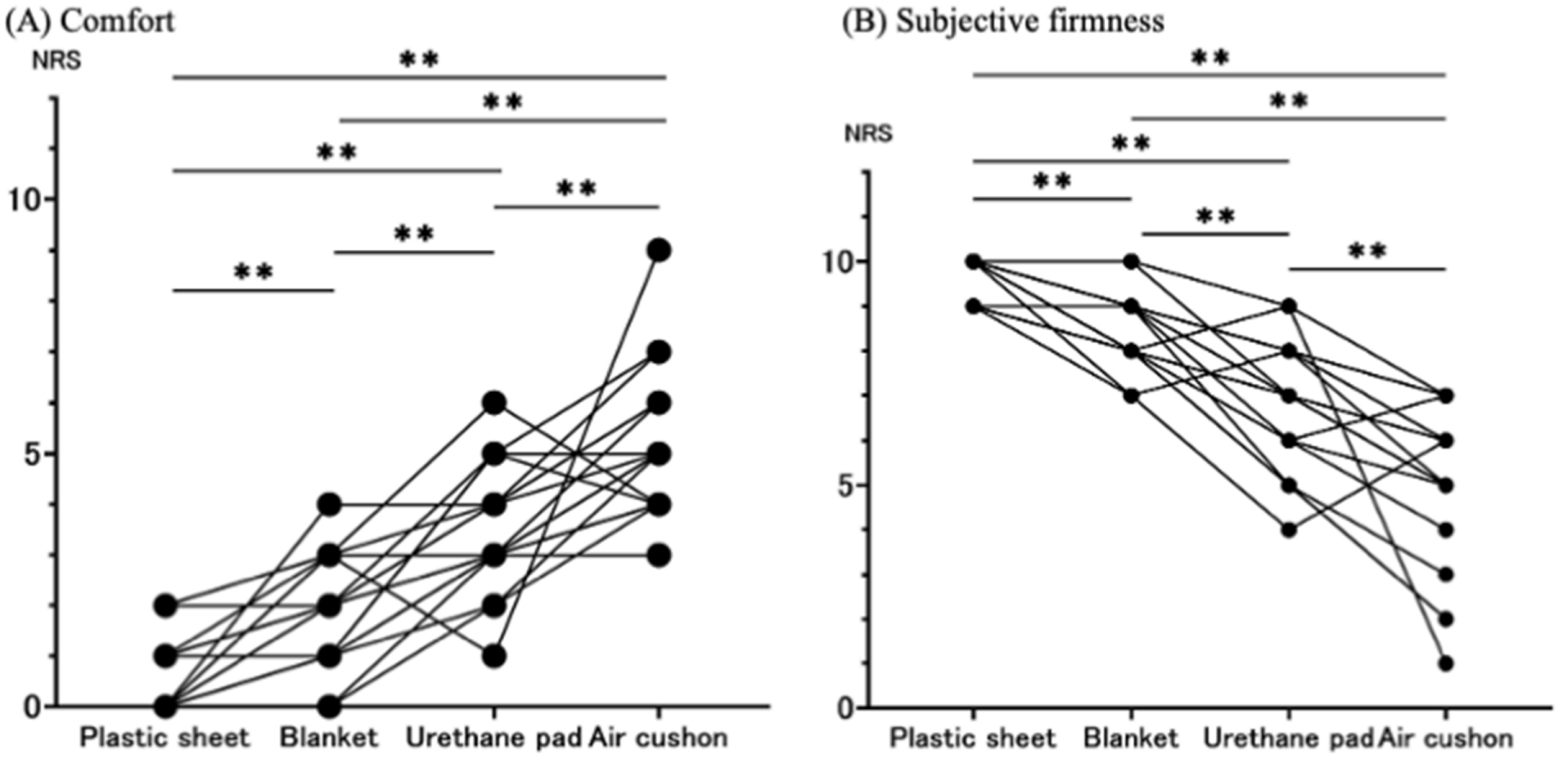
Subjective ratings of Firmness and Comfort. Each dot represents an individual data measurement, and lines connecting these dots indicate repeated measurements from the same participant. All data were compared using a Linear Mixed Effect Model. * P < 0.05, ** P < 0.01.

Comparisons of the average contact pressure and contour area among the four types of mats are shown in Figs 4 and 5. When air cushions were used instead of plastic sheets, the average body pressure decreased by 21.6% in the supine position and by 20.4% in the lateral position. The contour area also increased by 35.4% in the supine position and by 32.6% in the lateral position with the air cushion. Additionally, the air cushion resulted in the lowest subjective firmness and the highest comfort levels (Fig 6). Notably, when comparing urethane pads and air cushions, the air cushion yielded a significantly lower average body pressure (p < 0.01) and a significantly larger contour area (p < 0.01). In this study, we employed a partial crossover design for the third and fourth intervention periods, in which urethane pads and air cushions were used. No significant carryover effects were observed across all measured outcomes (S1 Table).

## Discussion

This study indicated that using air cushions as floor mats in evacuation shelters could improve the body pressure distribution among evacuees. Additionally, using air cushions also led to lower subjective firmness and greater comfort.

For many years, emergency beds were not provided for evacuees in Japanese evacuation shelters. However, since the Great East Japan Earthquake, cardboard beds have been introduced to mitigate various health issues faced by evacuees during large-scale disasters. Nevertheless, because Japanese municipalities are not required to stockpile these beds, evacuees are still forced to sleep on shelter floors for weeks or even months.

During winter disasters such as the Noto Peninsula Earthquake, cold temperatures could be a critical risk factor for the health of older adults and individuals with chronic illnesses [15]. However, many municipalities have not stockpiled floor mats other than plastic sheets with poor insulating properties in shelters. Therefore, prolonged exposure to the cold floors in evacuation shelters could pose significant health risks for evacuees. Our study suggested that 1.6-cm-thick air cushions have a similar insulating capacity to 2.0-cm-thick urethane mats used in camp. Therefore, providing these floor mats could help protect evacuees from cold exposure and maintain their health and comfort. However, because this study did not assess the extent to which the observed increase in insulation translated into improvements in thermal comfort, further research is required to quantify these effects.

It has been suggested that excessively firm bedding surfaces may also increase the risk of back pain and sleep problems [8,10,12,16]. In evacuation shelters where only plastic sheets are placed on the floor, body pressure is not adequately dispersed, potentially leading to back pain among evacuees. When air cushions were used, average body pressure decreased by more than 20%, and the contact area increased by over 30% compared with simply placing a plastic sheet on the floor. Further, it has been suggested that the 1.6-cm-thick air cushion could reduce mean body pressure and increase contour area to a degree comparable to the 5-cm-thick air mattress evaluated in a previous study [8].

Further, among all the conditions examined, the use of air cushions resulted in the lowest subjective firmness scores and provided the most comfortable experience. In evacuation shelters where only plastic sheets are used on the floor, body pressure is not adequately dispersed, potentially leading to back pain among evacuees. Our study also revealed that while the urethane pad provided slightly better thermal insulation, the air cushion offered superior body pressure distribution compared to the urethane pad.

Although cardboard beds can reduce some of the negative health consequences of floor sleeping, they tend to be heavy (approximately 10 kg) and relatively expensive, which limits their large-scale stockpiling by municipalities. In contrast, air cushions are inexpensive, lightweight, and can be stored compactly, making them more feasible for disaster preparedness. Since the surfaces of cardboard beds are as firm as the floor, the use of a mattress is necessary; however, air cushions can be used as a substitute for mattresses [8,12]. While the provision of cardboard beds to evacuees may take several months, stockpiling air cushions is expected to quickly improve evacuees’ quality of life (QOL) when disasters occur.

Although Japanese municipalities have stockpiled blankets for evacuees, our previous study showed that placing these blankets on the floor could not effectively distribute evacuees’ body pressure [12]. Conversely, the results of this study indicated that even blankets could help disperse body pressure to some extent. In the previous research, we used a dummy model instead of human subjects and a pressure-distribution measurement system that could not examine pressures above 100 mmHg. These differences in experimental conditions may have resulted in the divergent outcomes in both studies [12].

This study has several limitations. First, older adults and obese individuals are generally at higher risk of back pain; however, our sample had limited demographic diversity. Of the 20 participants, 19 (95%) were female, with a mean age of 23.5 years and a mean BMI of 20.3 kg/m². This homogeneous demographic profile does not adequately represent the general population. The significant gender imbalance, young age profile, and relatively low BMI restrict the generalizability of our findings to broader populations, particularly male individuals, older adults, or those with higher BMI values. Although we used a linear mixed model with sex and BMI as covariates, future studies should recruit larger and more demographically diverse samples to enhance external validity. Second, we used a body pressure measurement device with a maximum measurable contact pressure of 200 mmHg; therefore, pressures exceeding 200 mmHg were not recorded, and the actual average pressure might have been higher than reported. Third, the short duration of the intervention was a significant limitation. Because of the short duration, we could not assess the long-term benefits of air cushions in preventing musculoskeletal strain or improving sleep quality. Therefore, these potential benefits remain uncertain, and further research is needed to elucidate their effects. Fourth, we did not evaluate thermal comfort or body temperature changes associated with air cushion use. Future research should incorporate thermal comfort assessments and temperature monitoring to provide a more comprehensive understanding of the physiological responses to air cushions.

## Conclusions

This study demonstrated that air cushions provide effective body-pressure dispersion, reducing average contact pressure by more than 20% compared to plastic sheets alone, while maintaining superior insulating properties. These findings suggest that air cushions may offer a practical solution for alleviating musculoskeletal strain and pressure-related discomfort.

## Supporting information

S1 Table Results of the linear mixed-effect model. Data were analyzed using a linear mixed-effects model, with a Bonferroni correction applied for multiple comparisons. Effect estimates (with 95% CIs and p-values) are shown. For Intervention, air cushions and urethane mats were compared, and for Period, the 3rd and 4th sessions were compared. P-value of less than 0.05 was considered statistically significant.
(XLSX)

S1 Text CONSORT Checklist.
(DOC)

S2 Text Study Protocol.
(DOCX)
