# Supplementary material for: Evaluating the effects of air cushions on body pressure distribution and thermal insulation in evacuation shelters: A randomized controlled crossover study
Source: PLOS Glob Public Health. 2025 Oct 6;5(10):e0005259. doi: 10.1371/journal.pgph.0005259 (PMC12500142; doi:10.1371/journal.pgph.0005259)
Supplement: S2 Text — Study Protocol. (DOCX) [file pgph.0005259.s003.docx]

**Study Protocol**

**Study Title**

Evaluating the effects of air cushions on body pressure distribution and thermal insulation in evacuation shelters: A randomized controlled crossover study.

**Principal Investigator**

Seiji Hamanishi

**Research Institution**

Nursing Faculty, Kansai University of Social Welfare

**Study Objectives**

This study aimed to determine whether air cushions can provide sufficient body pressure dispersion for use as floor mats in evacuation shelters.

**Study Design**

This trial employs a randomized, controlled crossover design. In this study, all participants receive all interventions, but the order in which they receive them (the sequence) is randomized. All subjects are randomly assigned to one of two sequences (A or B) using random numbers generated in Microsoft Excel. Blinding is not employed in this trial.

**Inclusion Criteria**

1. People who aged between 18 and 60 years.

2. People who can read and fill out explanatory documents, consent forms, and survey forms written in Japanese.

**Exclusion Criteria**

1. People with difficulty assuming the postures required for the experiment.

2. People with a body mass index (BMI) less than 18 or greater than 30.

3. People having severe body pain.

**Intervention**

A: Plastic sheet

B: Blanket

C: Urethane pad

D: Air cushion

**Study Procedures**

**Sequence A**

Plastic sheet → Blanket → Urethane pad → Air cushion

**Sequence B**

Plastic sheet → Blanket → Air cushion → Urethane pad

10-minute washout period is inserted between each experimental period.

Body pressure distribution is measured twice for each mattress condition and the average value is calculated.

**Outcome**

**Primary outcome**

Body pressure distribution (the body contact pressure and the body surface contour area)

**Secondary outcome**

Subjective comfort and firmness (NRS)

**Other data**

Change in surface temperature when ice packs contact each sheet.

Patient information on individual attributes, including age, sex, and body mass index (BMI).

**Statistical analysis**

A linear mixed-effects model is used to examine the effect of air cushion on body pressure distribution. Period, sequence, and floor mat were entered into the model as fixed effects, and subjects are entered as random effects. A Bonferroni analysis is performed to compare the main effects. Age, sex, and BMI were entered into the model as covariates to control for potential confounding factors. In our study, a P-value < 0.05 is considered statistically significant.

**Ethics approval and consent to participate**

This study is performed with the approval of the Ethical review board of Kansai University of Social Welfare. The methods are carried out according to approved guidelines. All study participants provide informed written consent prior to study enrollment. This trial is registered in the University Hospital Medical Information Network Clinical Trials Registry (No.: UMIN000051487)　.
